# An updated overview of anticancer effects of alternariol and its derivatives: underlying molecular mechanisms

**DOI:** 10.3389/fphar.2023.1099380

**Published:** 2023-03-23

**Authors:** Muhammad Torequl Islam, Miquel Martorell, Carlos González-Contreras, Marcelo Villagran, Lorena Mardones, Bekzat Tynybekov, Anca Oana Docea, Ahmad Faizal Abdull Razis, Babagana Modu, Daniela Calina, Javad Sharifi-Rad

**Affiliations:** ^1^ Department of Pharmacy, Life Science Faculty, Bangabandhu Sheikh Mujibur Rahman Science and Technology University, Gopalganj, Bangladesh; ^2^ Department of Nutrition and Dietetics, Faculty of Pharmacy, University of Concepción, Concepción, Chile; ^3^ Centre for Healthy Living, University of Concepción, Concepción, Chile; ^4^ Universidad de Concepción, Unidad de Desarrollo Tecnológico, UDT, Concepción, Chile; ^5^ Biomedical Sciences Research Laboratory, Faculty of Medicine, Universidad Católica de la Santísima Concepción, Concepción, Chile; ^6^ Department of Biodiversity of Bioresources, Al-Farabi Kazakh National University, Almaty, Kazakhstan; ^7^ Department of Toxicology, University of Medicine and Pharmacy of Craiova, Craiova, Romania; ^8^ Department of Food Science, Faculty of Food Science and Technology, Universiti Putra Malaysia, Selangor, Malaysia; ^9^ Natural Medicines and Products Research Laboratory, Institute of Bioscience, Universiti Putra Malaysia, Selangor, Malaysia; ^10^ Department of Biochemistry, Faculty of Science, University of Maiduguri, Maiduguri, Nigeria; ^11^ Department of Clinical Pharmacy, University of Medicine and Pharmacy of Craiova, Craiova, Romania; ^12^ Facultad de Medicina, Universidad del Azuay, Cuenca, Ecuador

**Keywords:** alternariol, mycotoxin, cancer, molecular targets, cytotoxic effect, apoptosis, chemotherapy

## Abstract

Alternariol is a toxic metabolite of Alternaria fungi and studies have shown multiple potential pharmacological effects. To outline the anticancer effects and mechanisms of alternariol and its derivatives based on database reports, an updated search of PubMed/MedLine, ScienceDirect, Web of Science, and Scopus databases was performed with relevant keywords for published articles. The studies found to suggest that this mycotoxin and/or its derivatives have potential anticancer effects in many pharmacological preclinical test systems. Scientific reports indicate that alternariol and/or its derivatives exhibit anticancer through several pathways, including cytotoxic, reactive oxygen species leading to oxidative stress and mitochondrial dysfunction-linked cytotoxic effect, anti-inflammatory, cell cycle arrest, apoptotic cell death, genotoxic and mutagenic, anti-proliferative, autophagy, and estrogenic and clastogenic mechanisms. In light of these results, alternariol may be one of the hopeful chemotherapeutic agents.

## 1 Introduction

Chemotherapy is a type of anticancer treatment that uses one or more chemical substances (extracts from natural substances or products of chemical synthesis) that stop the multiplication of cancer cells, either by destroying them or by stopping their division. Chemotherapy is an essential component of the pharmacotherapeutic management of cancer. (Sharifi-Rad et al., 2021b; GBD, 2019 Colorectal Cancer Collaborators, 2022). On the other hand, the resistance of cancer cells towards chemotherapeutic drugs has become prevalent, being associated with unfavourable clinical evolution in cancer patients directly and indirectly (Sharifi-Rad et al., 2021e; Sharma et al., 2022). The progress of new drug research and development may overcome the occurrence of drug resistance (Ali et al., 2022b). It has been also reported that natural products and derivatives with diverse chemical structures and pharmacological effects serve as useful compounds against cancer and drug-resistant cancer (Ali et al., 2022a; Dhyani et al., 2022b; Kitic et al., 2022; Sharifi-Rad et al., 2022a). Mycotoxins are types of toxins produced by a variety of fungal species of crops or stored commodities. Mycotoxins appear as primary or secondary contaminants *via* the carryover effect in the food chain (Degen, 2017). Due to various biological effects, mycotoxins have come to the attention of scientists in the context of research done to discover and develop new anticancer drugs (Islam, 2017; Islam et al., 2018). Generally, mycotoxins are ubiquitous and unavoidable harmful fungal products and vary significantly in structure and biochemical effects. These toxins cause disease in both animals and humans and are found in almost all types of foods, with a higher prevalence in hot, humid environments (El Khoury et al., 2019). Unfortunately, most of the published data has concerned the major mycotoxins aflatoxins, ochratoxin A, zearalenone, fumonisins and trichothecenes, especially deoxynivalenol (Smith et al., 2016), although, there are aspects of mycotoxin relations with strain improvement strategies and genetic modification for improved detoxifying properties in test systems (Pfliegler et al., 2015).

Alternariol (AOH), a toxic mycotoxins metabolite of *Alternaria* fungi, is an essential contaminant in cereals and fruits. Alternaria fungi are plant and human pathogens, saprophytes, a strong allergen and exposure has been associated with allergic diseases such as allergic rhinitis, chronic rhinosinusitis and asthma (Grover and Lawrence, 2017; Aichinger et al., 2021). Mycotoxins enter the body through contaminated food, but can also enter the airway or through direct skin contact. In general, mycotoxins are resistant to high temperatures, and many mycotoxins are also resistant to industrial food processing, so to have mycotoxin-free foods, the raw material (wheat, milk, vegetables, meat, etc.) must be analyzed. Because they are resistant to processing, they can also be found in highly processed foods such as bread, breakfast cereals, wine, and beer. Many pharmacological activities, including antifungal (da Cruz Cabral et al., 2019), anti-inflammatory (Kollarova et al., 2018), and anticancer effects have been done (Meena and Samal, 2019). This updated review sketches a current scenario of AOH’s anticancer effect and possible action mechanisms behind it based on database information.

## 2 Review methodology

A literature study was conducted up to December 2021 using the following databases: PubMed/MedLine, Science Direct, Web of Science, Scopus, and the American Chemical Society using the next MeSH terms: “Alternariol,” “Alternariol monomethyl ether,” “Alternaria/metabolism,” “Mycotoxins,” “Cell Line,” “Tumor,” “Cell Survival/drug effects,” “Humans,” “Mycotoxins/toxicity,” “Reactive Oxygen Species.” No language restrictions were imposed. Articles were evaluated in detail and summarized information on the dose, concentration, administration route, experimental model, results discussion, conclusion, and the proposed action mechanism.

### 2.1 Inclusion criteria

1. Pharmacological studies carried out *in vitro*, *in vivo* with or without using experimental animals, including humans and their derived tissue and cells
2. Studies with AOH and its derivatives and joint effects with other substances (including drugs or chemicals/biochemicals)
3. Studies with or without proposing activity mechanisms.

### 2.2 Exclusion criteria

1. Studies with extracts without phytochemical analysis
2. Studies with homeopathic drugs
3. Other studies of AOH uncover the current topic.

## 3 Stability, bioavailability and pharmacokinetics

A recent study showed a significant reduction in AOH when exposed to a temperature of 35°C, and very high temperatures above 100°C significantly affect its stability. Compared to this, its derivative, alternariol monomethyl ether (AME) is much more stable; at high temperatures of 80°C–110°C (Estiarte et al., 2018). AOH suffers several reactions of biotransformation, as has been demonstrated by studies performed *in vivo*, in rat liver slices, cell culture or purified enzymes. The identified chemical modifications of AOH include hydroxylation (phase I biotransformation), sulfation and glucuronidation (phase II biotransformation), which are executed mainly by cytochrome P450 isoforms (Tran et al., 2020). The principal organ of AOH metabolization is the liver, although other organs like the kidneys, the bladder and components of the gastrointestinal tract have also been involved. Of note is the lack of relevant participation of gut microbiota in the biotransformation of AOH (Lemke et al., 2016). Some enzymes responsible for AOH metabolization are uridine 5′-diphosphate-glucuronosyltransferase, glutathione S-transferase and CYP1A1 (Appel et al., 2021). The last one is responsible for hydroxylation at C-2, C-4, and C-8. Subsequently, 4-hydroxy-AOH is glycosylated to 3-glucoside (58%) and 9-glucoside (5%) in the whole-cell system. The metabolite 9-diglucoside can also be hydrolyzed to 9-glucoside. Some metabolites of AOH, as catechols formed by its hydroxylation, can also be methylated and hydroxylated.

*In vivo* studies performed in rodents reveal that a high percentage (85%–91%) of AOH given orally is excreted in the faeces, and a low percentage in the urine (>2.6%), with 0.8% urine excretion of alternariol-3-sulphate (Schuchardt et al., 2014). The blood concentration of AOH only reaches 0.06% after 24 h when 2000 mg/kg are administrated orally. However, when doses were applied in triplicate at 0, 24 and 45 h, AOH reached a blood concentration of 0.5 µM after 3 h of administration. The study performed by Schuchardt et al. (2014), could detect 4, 10, 8, and 2 hydroxy metabolites of AOH in urine the following three days after the triple doses. Using polarized human colon adenocarcinoma Caco-2 cells culture as a model of the intestinal barrier, it has been established that between 23% and 26% of the apically applied AOH crosses the cell barrier, founding several metabolites on the basolateral side (Appel et al., 2021). When CaCo-2 cells were cultured with AOH or 9-glucoside alternariol, a similar distribution of derivates were found on the apical and basolateral side. Particularly, after 3 h of apical exposure, 45% of the initial compounds of the supernatant corresponded to 9-glucoside, 15% to 3-glucuronide, 14% to 3-sulphate and 11% to 9-glucuronide. Using AOH unconjugated molecule, there was 8% of the recovered compounds. Specifically, in the cell, the glucuronides and the glucoside also could be detected. The 3-glucoside plant metabolite displays a different distribution in the whole cell system, showing over 90% of metabolites recovered. Also, in the cells, only traces of the same metabolites were detected, including the unmodified AOH. On the other hand, 9-diglucoside has no significant absorption. In summary, the glucuronides and sulfates of AOH showed moderate absorption (20%–70%), meanwhile, the free mycotoxin and the 9-glycoside have higher absorption (Burkhardt et al., 2009). These data reveal that AOH and its metabolites are significatively absorbed by epithelial cells, but the localization of the glycosylation position affects its absorption and metabolization.

## 4 Anticancer mechanisms and targeting signaling pathways by alternariol

### 4.1 Cytotoxicity

Cytotoxic effect test for an anticancer agent is the first option as it tells whether the agent should be considered an anticancer drug or not (Zlatian et al., 2015; Docea et al., 2016). In this context, time and concentration-dependent cytotoxic effect measurements are crucial (Docea et al., 2012; Sharifi-Rad et al., 2021c). Due to its cytotoxic properties, AOH could be a good candidate for exploring anticancer effects (Figure 2). This possibility was evaluated in a recent study by Palanichamy et al. (2019) where human hepatocarcinoma cells (HUH-7), and human alveolar epithelial cells (A549) were exposed to purified AME for 48 h. HUH-7 cells were the most sensitive to the cytotoxic effect, with an IC_50_ of 50 μM and showing a cell cycle arrest at the G1 phase. Within the same study, AME was able to protect from neoplastic transformation induced by diethylnitrosamine in rat livers.

AOH and alternariol monomethyl ether (AME) are evident to show strong cytotoxic effect (IC_50_ values of 3.12–3.17, and 4.82–4.94 μg/mL), while AOH derivative, alternariol 4-methyl-10-acetyl ether, and alternariol 3,9-dimethyl ether exhibited weak activities (IC_50_ values > 50 μg/mL) against human epidermoid carcinoma (KB and KBv200) cell lines (Tan et al., 2008). AOH (3.125–100 μM) was found to exert cytotoxic effects in CaCo-2 cells (Vila-Donat et al., 2015). In another study, AOH (12.5–100 µM) was found to augment reactive oxygen species (ROS) generation and eventually exert a cytotoxic effect in CaCo-2 cells (Chiesi et al., 2015). Moreover, AOH and AME at 3.125–100 µM exerted cytotoxic and combined cytotoxic effects in CaCo-2 cells (Fernández-Blanco et al., 2016).

### 4.2 Induced oxidative stress in cancer cells

Chemotherapeutic agents act through many pathways (Mitrut et al., 2016; Hossain et al., 2021). Chronic ROS induction and mitochondrial dysfunction-linked exerting a cytotoxic effect are one of them (Sharifi-Rad et al., 2020; Scheau et al., 2021). Therefore, the regulation of oxidative stress is an essential factor in anticancer therapies (Sharifi-Rad et al., 2021a; Sharifi-Rad et al., 2021c). AOH (25–200 µM) caused ROS generation, leading to mitochondrial dysfunction-dependent cytotoxic effect in human colon carcinoma (HCT116) cells (Bensassi et al., 2012). AOH-induced ROS production and an increase in cellular stress were also evident in RAW264.7 macrophages (Solhaug et al., 2012; Solhaug et al., 2014), and CaCo-2 cells (Fernández-Blanco et al., 2014; Fernández-Blanco et al., 2015). In another study, AOH and AME at 0.1–50 µM modulated the redox balance of HT29 cells (human colon cancer cell line), but without apparent adverse effect on DNA integrity (Tiessen et al., 2013).

### 4.3 Effects on inflammation and immunity

There is a relationship between inflammation and cancer (Jain et al., 2021). Chronic inflammation can induce tumorigenesis by initiating and perpetuating local inflammatory processes that promote the proliferation and dissemination of tumor cells. Therefore, inflammatory pathways may be targeted by alternariol in an attempt to control cancer (Ali et al., 2022a; Hossain et al., 2022; Iqbal et al., 2022).

MAPK mitogen-activated protein kinase (MAPK) pathway is vital for the adaptation of the cell to stress and its activation is highly involved in the inflammation process (Motyka et al., 2023; Prasher et al., 2023). The cell inflammation induction by lipopolysaccharide (LPS) triggers a series of signaling pathways including MAPK and nuclear factor kappa-light-chain-enhancer of activated B cells (NF-κB) (Li et al., 2016; Capó et al., 2021; Pezzani et al., 2023). MAPKs are involved in the phosphorylation of JNK, ERK and p38 which regulate the expression of MSK 1/2 and then p65 (Xie et al., 2019; Garzoli et al., 2022; Li et al., 2023). Inducible nitric oxide synthase (iNOS) and cyclooxygenase-2 (COX-2) are also key enzymes involved in inflammation and cell stress, being NO an important regulator of COX-2 expression and activity (Sharifi-Rad et al., 2022c; Li et al., 2023). AOH showed to lead to the phosphorylation of cAMP response element-binding (CREB) and increased the expression of COX-2 (Bansal et al., 2019).

AOH (12.5–50 µg/animal (single topical) in mice showed dermal toxicity by activating the EP2/cAMP/p-CREB signaling cascade (Bansal et al., 2019). In this study, an increase in bi-fold thickness, as well as hyperplasia and higher production of prostaglandin E2 (PGE2) along with cyclic adenosine monophosphate (cAMP), COX-2, cyclin D1 as well as prostanoid EP2 receptor in the skin, was also seen. Moreover, AOH (1–20 µM) showed to suppress the LPS-induced NF-κB pathway activation, decreased the secretion of the proinflammatory cytokines interleukin (IL)-8, IL-6, tumor necrosis factor-alpha (TNF-α) and induced IL-10 secretion (Kollarova et al., 2018). In the latter case, a dose-dependent downregulation of miR-146a while upregulation of miR-155 was also seen in THP-1-derived macrophage cells. AOH and AME have been reported to counteract pro-inflammatory stimuli in different cell models (Grover and Lawrence, 2017; Kollarova et al., 2018; Schmutz et al., 2019; Aichinger, 2021). The hematology and serum biochemistry results obtained in a study performed in male Sprague-Dawley rats showed that the administration of AME (1.84, 3.67, or 7.35 μg/kg body weight/day) for 28 days compromises the immune system (Tang et al., 2022). The suggested mechanisms involved are the cholesterol-like intercalation into the cell membranes of macrophages (Del Favero et al., 2020) and the interaction with NF-κB signaling mediated *via* Nrf2 activation (Khandia et al., 2019).

### 4.4 Cell cycle arrest

All cells that multiply do so through what is known as the cell cycle (Asgharian et al., 2022; Sharma et al., 2022). The cell cycle is a succession of carefully controlled phases so that if things do not go well in a certain phase (for example, genetic alterations occur), the cell cannot progress to the next phases of the cycle (Javed et al., 2022; Chaudhary et al., 2023). In cancer, these checkpoints are disrupted (Ianoși et al., 2019; Limas and Cook, 2019; Dhyani et al., 2022a). Some of the drugs and bioactive natural compounds used in cancer treatment, can restore normal signaling and control pathways or disrupt the activity of signaling and control pathways that no longer function normally (Salehi et al., 2019; Sharifi-Rad et al., 2022b; Taheri et al., 2022). In porcine endometrial cancer cells, AOH (0.39-15.5 µM) decreased cell number and reduced cells in the S phase together with the arrest of the cells in the G0/G1 phase (Wollenhaupt et al., 2008). It is also found to cause abnormal nuclear morphology and cell cycle arrest at the G2/M phase in RAW 264.7 macrophage cells (Solhaug et al., 2013; Solhaug et al., 2014).

### 4.5 Apoptosis of cancer cells

Apoptosis is a form of programmed cell death that occurs in our body in which many intrinsic and extrinsic events lead to characteristic cell changes (morphology) and death (Amin et al., 2022; Irfan et al., 2022; Javed et al., 2022). Many anticancer drugs are evident to enhance this process of cell death (Sharifi-Rad et al., 2021b; Sharifi-Rad et al., 2021e; Dhyani et al., 2022b). AME (25–200 µM) induced cell death in human colon carcinoma (HCT116) cells by activating the mitochondrial pathway of apoptosis (Bensassi et al., 2011). In the same cell line, AOH induced apoptosis *via* the mitochondria-dependent pathway, characterized by a p53 activation, an opening of the mitochondrial permeability transition pore (PTP), triggering a loss of mitochondrial transmembrane potential (DWm), and a downstream generation of anion superoxide and caspase −9 and −3 activation (Bensassi et al., 2012). In addition, the deficiency of the pro-apoptotic protein Bax was also observed in this study. AOH (20 µM) and AME (40 µM) were found to induce CYP1A1 and cause apoptotic cell death in murine hepatoma (Hepa-1c1c7, Hepa-1c1c4) cells (Schreck et al., 2012).

### 4.6 Genotoxicity and mutagenic effects

Anticancer drugs can also act by exerting genotoxic and mutagenic effects on cancer cells. These are also reported as cytotoxic mechanisms (Buga et al., 2019; Islam et al., 2021; Asgharian et al., 2022). An earlier report suggests that AOH inhibited DNA strand breakage in an *in vitro* model (Xu et al., 1996). AOH (15–30 µM), in RAW 264.7 cells, caused DNA damage *via* phosphorylation of histone H2AX and checkpoint kinase (Chk-1/2). Activated p53 and increased the expression of p21, Cyclin B, MDM2, and Sestrin 2 likewise, the level of several miRNAs was also affected (Solhaug et al., 2012). AOH, AME, and altertoxin II (0–20 µM) caused DNA strand breaking and showed a mutagenic effect in cultured Chinese hamster V79 cells (Fleck et al., 2012). In this study, altertoxin II was more potent than AOH and AME. AOH, in RAW264.7 macrophage cells, caused DNA damage (double-strand breakage) (Solhaug et al., 2014). In a recent study, AOH and altertoxin II have been also evident to cause DNA damage and exert genotoxic effects in nucleotide excision repair-deficient cells (Fleck et al., 2016). AOH (10 µM) was found to exert mutagenic effects in V79 and mouse lymphoma L5178Y tk ± cells (Brugger et al., 2006). Moreover, in a molecular docking study, AOH and AME were found to disrupt topoisomerases and lead to genotoxic outcomes (Dellafiora et al., 2015).

### 4.7 Anti-proliferative effect

Cancer is characterized by the uncontrolled proliferation of abnormal cells (Salehi et al., 2021; Sharifi-Rad et al., 2021d). AOH exerted an anti-proliferative effect in CaCo-2 cells (Vila-Donat et al., 2015). AOH is also evident to exert an anti-proliferative influence on RAW 264.7 (Solhaug et al., 2012) and CaCo-2 cells (Fernández-Blanco et al., 2014). AOH also inhibited cell proliferation by interfering with the cell cycle in Ishikawa and V79 cells (Lehmann et al., 2006).

### 4.8 Autophagy

Anticancer drugs can induce autophagy in cancer cells (Sani et al., 2017). In a study, of RAW264.7 macrophage cells when treated with AOH (15–60 µM) the autophagy marker LC3 was markedly increased (Solhaug et al., 2014). In this study, activation of p53 and the Sestrin2-AMPK-mTOR-S6K signaling pathway was also seen.

**FIGURE 1:**
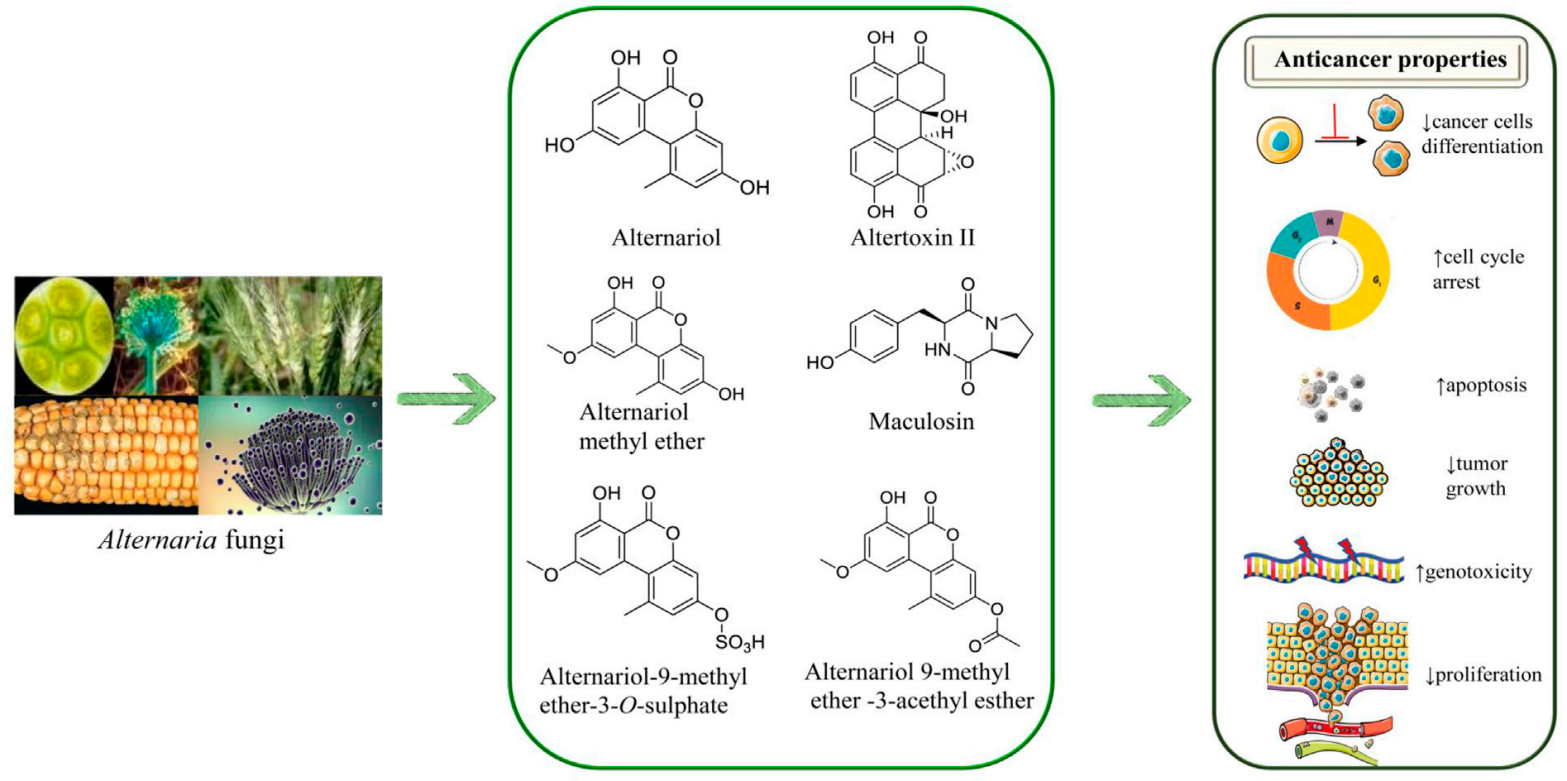
The chemical structures of Alternariol and its derivatives and their anticancer potential mechanisms. Symbols: ↑ (increased), ↓(decreased).

Anticancer effects of AOH and/or its derivatives from *in vitro* studies have been shown in Table 1. The chemical structures of AOH and its most representative derivatives are represented in Figure 1.

**TABLE 1:** Anticancer effects of alternariol and/or its derivatives in different *in vitro* experimental studies.

AOH/Derivatives	*in vitro* cell Lines/IC_50_	Potential anticancer mechanisms	Ref.
AOH	colon cancer cells	AOH 65 μM	Bensassi et al. (2012)
	HCT116	↑apoptosis	
	HCT116 Bax-KO	- ↑ROS, ↓O_2_ radicals	
	(HCT116 deficient for Bax cells)	- ↑mitochondrial PTP	
	IC_50_ = 25–200 µM	- ↓DWm	
	control: H_2_O_2_1 mM	AOH 50 μM	
		↑apoptosis, ↑caspase-3, ↑caspase-9, ↑ p53, ↑DNA damage, ↑Bax, ↑mitochondrial permeabilization ↓ionic homeostasis ↑membrane rupture	
		↑death-promoting factors cytC, EndoG	
	colon carcinoma cells	AOH 3,125 μM	Vila-Donat et al. (2015)
	CaCo-2	↑LPO, ↑ROS, ↑oxidative stress, ↑cytotoxicity	
	IC_50_ = 3.125–100 μM	AOH 50–100 µM	
	control: 1% DMSO	↓cell proliferation	
	colon carcinoma cells	↓ROS	Chiesi et al. (2015)
	CaCo-2	↑cytotoxicity	
	IC_50_ = 12.5–100 µM	↓cells viability	
	control: 1% DMSO		
	murine macrophage cell lines	AOH 30 μM	Solhaug et al. (2014)
	RAW 264.7	↑ROS, ↑cellular stress, ↑cell cycle arrest, ↑ autophagy, ↑ senescence, ↑DNA damage, ↑p53 ↑topoisomerase, ↑ Sestrin2-AMPK-mTOR-S6Ks	
	IC_50_ = 15–60 µM	↑abnormal nuclear morphology,	
	positive control: salt solution (EBSS)	↑vacuolization of the cytoplasm	
	colon carcinoma cells	↑ROS	Fernández-Blanco et al. (2014)
	CaCo-2	↑oxidative stress	
	IC_50_ = 3.125–100 µM	↓ cancer cells proliferation	
	control: 1% DMSO		
	porcine endometrial cells	↓cells number	Wollenhaupt et al. (2008)
	IC_50_ = 0.39–15.5 µM	↓cells in the S phase	
	control: 1% DMSO	↑arrest of the cells in the G0/G1 phase	
	neoplastic Chinese hamster cell lines	DNA strand breakage	Fleck et al. (2012)
	V79	↑cell cycle arrest in the G2/M phase	
	IC_50_ = 0.1–5 µM	↑HPRT gene mutations	
	control: 1% DMSO		
	recombinant yeast	↑androgenic response	Stypuła-Trębas et al. (2017)
	*Saccharomyces*cerevisiae strains		
	EC_50_ = 269.4 μM.		
	control: 1% DMSO		
AOH	murine hepatoma cells	↑ CYP1A1	Schreck et al. (2012)
AME	Hepa-1c1c7	↑apoptosis	
	Hepa-1c1c4	↓ cell numbers	
	Hepa c1c12		
	IC_50_ = 20–40 µM		
	control: 0.4% DMSO		
	human colorectal cancer cell line	↑cytotoxicity	Tiessen et al. (2013)
	HT29	↑intracellular redox status, ↑Nrf2, ↑GSH, ↑GST, ↑oxidative DNA-damage	
	IC_50_ = 0.1–50 µM	↑Nrf2/ARE-dependent gene transcription	
	control: 1% DMSO		
	CaCo-2 cells	↑ cytotoxicity, ↓ cell viability	Fernández-Blanco et al. (2016)
	IC_50_ = 3.125–100 µM	↓CaCo-2 cells growth	
	control:1% DMSO	AOH + AME→ ↑cytotoxicity effect	
AOH, AME, alternariol 4-methyl-10-acethyl ether alternariol 3,9-dimethyl ether	human oral squamous carcinoma cell line KB	↑cytotoxicity	Tan et al. (2008)
	multiple-drug resistant human oral squamous cells KBv200		
	IC_50_ = 3.12–3.17 μg/mL		
	IC_50_ = 82–4.94 μg/mL		
	control: 0.1% DMSO		
AOH	neoplastic Chinese hamster cell lines	↑DNA damage	Fleck et al. (2016)
Altertoxin II	V79	↑genotoxicity	
	hepatocellular carcinoma cell lines		
	HepG2		
	Nucleotide excision repair-deficient cells		
AOH, AME	neoplastic Chinese hamster cell lines	AOH 0.75 μM:	Fleck et al. (2012)
Altertoxin II	V79	↑mutagenic effect	
	IC_50_ = 0–20 µM	↑HPRT mutation, ↑DNA damage	
	positive control: salt solution (EBSS)	altertoxin II was more potent than AOH and AME	
AME	human colon carcinoma cells	↑cell death	Bensassi et al. (2011)
	HCT116	↑apoptosis	
	IC_50_ = 25–200 µM		

**Abbreviations and symbols**: ↑ increased, ↓ decreased, AIF, apoptosis-inducing factor; AME, alternariol monomethyl ether; AOH, alternariol; CYP, cytochrome c, DMSO, dimethylsulfoxide; DWm, mitochondrial transmembrane potential; EBSS, Earle’s balanced salt solution, EndoG endonuclease G, GSH, glutathione; GST, glutathione transferase; HPRT, hypoxanthine guanine phosphoribosyl transferase; LPO, lipid peroxidation; NQO, 4-nitroquinoline-N-oxide, Nrf2 nuclear factor erythroid 2-related factor 2, PTP, mitochondrial permeability transition pore, and ROS, reactive oxygen species.

**FIGURE 2:**
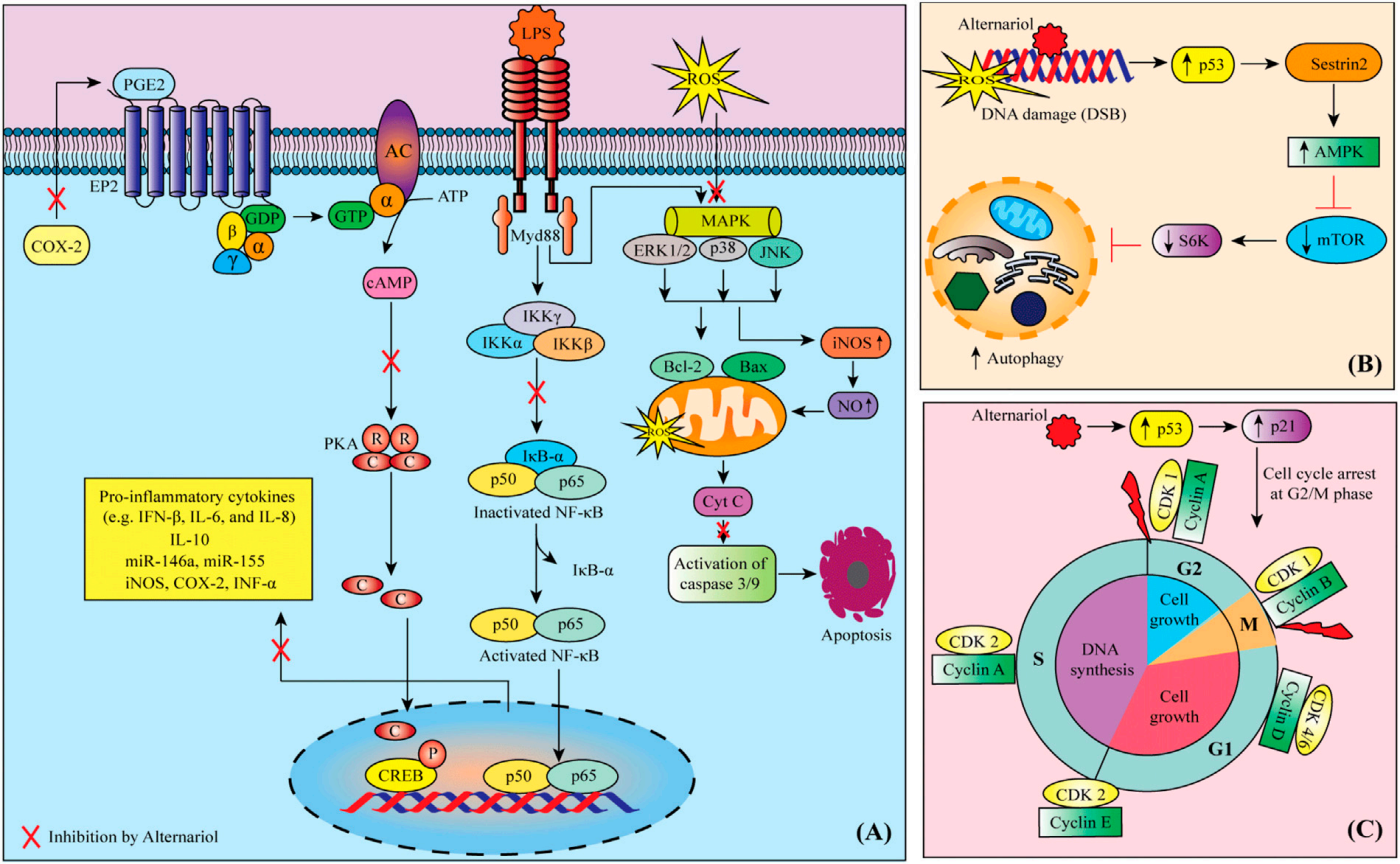
Possible mechanisms of anti-cancer activity of alternariol: **(A)** Alternariol induces apoptosis through targeting multiple deregulated signaling pathways in cancer cells, **(B)** Possible autophagy mechanism of alternariol through the activation of Sestrin2-AMPK-mTOR-S6K signaling pathway, **(C)** alternariol moderates the activity of cyclins and cyclin-dependent kinases to induce cell cycle arrest at G2/M phase. Abbreviations and symbols: ↑ increased, ↓ decreased, CDK cyclin-dependent kinase, COX-2 cyclooxygenase-2, CREB cAMP response element-binding, IFN interferon, IL interleukin, iNOS inducible nitric oxide synthase, LPS lipopolysaccharide, MAPK mitogen-activated protein kinase, mTOR mammalian target of rapamycin, PGE2 prostaglandin E2, PKA protein kinase A, ROS reactive oxygen species.

### 4.9 Other effects

AOH (0–10 µM) showed estrogenic and clastogenic potential, where replacement of E2 from human estrogen receptors α and β and increased the transcription of alkaline phosphatase (ALP) and its enzymatic activity in Ishikawa and V79 cells (human endometrial adenocarcinoma cell lines) (Lehmann et al., 2006). In this study, AOH also induced kinetochore-negative micronucleus in both cell lines. AOH and AOH derivatives, such as AME, alternariol-9-methyl ether-3-*O*-sulphate, and maculosin in leukemia, colon, lung and liver cancer cell lines, showed an efficient anticancer activity against leukemia, colon, lung and liver cancer cells (Hawas et al., 2015).

AOH (0.1–1000 ng/mL) in steroid hormone receptors, oestrogens androgens, progestagens, glucocorticoids and the H295R steroidogenesis assay, exhibited a weak oestrogenic response and binding of progesterone to the progestagen receptor was shown to be synergistically increased in the presence of AOH (Frizzell et al., 2013). In this study, was not observed a significant change in testosterone and cortisol hormones, but a significant increase in estradiol and progesterone production. Only one gene NR0B1 was downregulated, whereas expression of mRNA of CYP1A1, MC2R, HSD3B2, CYP17, CYP21, CYP11B2 and CYP19 was upregulated. On the other hand, in yeast estrogen and androgen reporter bioassays, AOH induced a full androgenic response in this eukaryotic test system (EC_50_ of 269.4 μM) (Stypuła-Trębas et al., 2017).

## 5 Toxicology and safety data

The toxicity of AOH has been studied since the 70s, mainly through *in vitro* models (Pero et al., 1973). However, insufficient *in vivo* data has prevented the assessment of AOH health risks for different species, including humans (EFSA Panel on Contaminants in the Food Chain et al., 2019). Among the first *in vivo* data, it is reported that toxins from *Alternaria* cultures are lethal when injected intraperitoneally at a dose of 100 mg/kg per day in DBA/2 mice (Pero et al., 1973). AOH is one of the 70 mycotoxins present in the *Alternaria* culture and produces itself, a median lethal dose of 400 mg/kg of body weight when administered in isolation in DBA/2 mice (Woody and Chu, 1992).

Another *in vivo* study aimed to study the genotoxic potential of AOH administered by oral gavage in NMRI mice (Schuchardt et al., 2014). The oral AOH did not cause an effect on the general health status during 7 days of observation, at doses up to 2000 mg/kg. Of note, the lack of toxicity could be related to the low systemic absorption of AOH, which reached blood levels of 0.5 µM representing less than 0.06% of the administered dose. At this low systemic concentration, AOH was negative for bone marrow micronuclei test and alkaline comet assay in the liver but the assays to investigate local genotoxicity in gastrointestinal tissues failed due to adverse effects of the AOH vehicle (corn oil) (Schuchardt et al., 2014).

A recent study investigated the effect of AOH in early-stage embryonic development through the injection of pregnant mice with AOH for 4 days. The highest dose of 5 mg/kg body weight/day caused injurious effects on embryonic development from the zygote to the blastocyst stage and also embryo degradation. Additionally, AOH also provoked a redox to unbalance in the offspring of mice during early gestation, suggesting that the toxin could act through an epigenetic mechanism (Huang et al., 2021). The reproductive and developmental toxicity of AOH could be related to its ability to act as an estrogen agonist. In this regard, AOH is a diphenolic compound that has some structural similarities to estrogen molecules and acts as a weak estrogen agonist as revealed by reporter assays in H295R cells (Frizzell et al., 2013). However, in other estrogen-responsive cells, like porcine granuloma cells, AOH failed to activate estrogen receptor a (Tiemann et al., 2009). In contrast to the effect on embryonic development in mammals, the injection of AOH into the yolk sac did not cause mortality or teratogenic effect in chicken embryos at doses up to 1 mg per egg (Griffin and Chu, 1983).

There is broader evidence regarding AOH toxicity *in vitro* models, including studies performed in bacterial strains and mammalian cell lines that show genotoxic activity (Solhaug et al., 2016). In *Salmonella* strains, AOH induces direct-acting AT base pair mutagenicity (Schrader et al., 2006). Also, its capacity to induce frameshift mutations was probed in *Bacillus subtilis* and *Escherichia coli* ND160 (EFSA Panel on Contaminants in the Food Chain et al., 2019). In mammalian cell lines, it has been reported that 1-h exposure to AOH in the range of 5–10 µM causes DNA strand breaks in V79 fibroblasts from Chinese hamsters, HepG2 hepatoma cells and HT-29 colon cells (Pfeiffer et al., 2007). Along with the mutagenic effect, AOH is also responsible for chromosome aberrations that are evident after 48 h of 10 µM exposure to the mycotoxin. Particularly, AOH induces kinetochore-negative micronuclei in Ishikawa and V79 cells, unscheduled DNA synthesis in the primary culture of human amniotic cells and increased mutations at the hypoxanthine phosphoribosyltransferase 1 (HPRT) gene in V79 fibroblasts (Lehmann et al., 2006). Another line of evidence suggests that DNA damage at molecular and chromosome levels is mediated by ROS production induced by AOH (Solhaug et al., 2016). This proposed mechanism is based on cytotoxicity assays of AOH, performed in cell lines including HT29, V79, RAW264.7 and Caco2 (Solhaug et al., 2012; Tiessen et al., 2013; Fernández-Blanco et al., 2014). For example, when Caco2 cells are exposed to AOH in a range of 15–60 µM for 24 h, there is a significant increase in ROS species, lipid peroxidation and a decrease in catalase and superoxide dismutase activities. Despite its oxidative effect, AOH produced a minor reduction in cell viability on Caco2, even at doses of 100 µM for 72 h (Fernández-Blanco et al., 2014).

Tiessen et al. (2013) also reported that AOH and AME induce an oxidative response in HT29 cells, including a transient decrease in glutathione levels, with a short exposure of 1 h. However, this effect did not produce DNA damage probably due to the activation of the redox-sensitive transcriptional response elicited by the transcription of Nrf2 (Tiessen et al., 2013). Another proposed mechanism for AOH genotoxicity is related to the inhibition of DNA topoisomerase I and IIa (Fehr et al., 2009). This enzyme is important to resolve topological constraints during DNA replication and therefore, it is likely that AOH-induced inhibition of topoisomerases could be responsible for the clastogenic effects observed in cell lines.

## 6 Limitations

Therapeutic limitations derive from insufficient knowledge of the pharmacokinetics, solubility, bioavailability, metabolism of alternariol, insufficient understanding of the molecular targets of action at the tumour cellular level, and their regulatory pathways. Although only experimental *in vitro* pharmacological studies have demonstrated and justified the anticancer effects of alternariol, translational pharmacological studies establishing the effective anticancer dose in humans, as well as clinical studies in humans, are lacking. Also, the development of new nanoformulations of alternariol in which it can be incorporated into different nanocarriers at the target should be the focus of future research. As a result, alternariol cannot be used in anticancer therapy as a first-line treatment, but only as an adjuvant in combination with standard chemotherapeutic treatment.

## 7 Concluding remarks

AOH and its derivatives, such as AME, alternariol-9-methyl ether-3-O-sulphate, alternariol 3,9-dimethyl ether and altertoxin II, exhibit an anticarcinogenic effect through several pathways, with ROS generation leading to the induction of oxidative stress and a cytotoxic effect linked to mitochondrial dysfunction, inflammatory pathway, cell cycle arrest in G0/G1, G2/M and S phases, apoptotic cell death, genotoxic and mutagenic mechanisms, antiproliferative, autophagy, as well as estrogenic and clastogenic mechanisms. To our knowledge, no other studies have explored the anticarcinogenic effect of AOH or its metabolites in animal models or clinical trials. This was corroborated by a search of the literature and also of US and European databases for completed or ongoing clinical trials (www.clinicaltrail.gov, www.clinicaltrailregister.eu). Given these promising results of experimental pharmacological studies, AOH and its derivatives can be considered potential adjunctive chemotherapeutic agents.

## Glossary

AIF: apoptosis-inducing factor
ALP: alkaline phosphatase
AME: alternariol monomethyl ether
AOH: alternariol
cAMP: cyclic adenosine monophosphate
CDK: cyclin-dependent kinase
Chk-1/2: checkpoint kinase
COX-2: cyclooxygenase-2
CREB: cAMP response element-binding
CYP: Cytochrome c
DMSO: dimethylsulfoxide
DWm: mitochondrial transmembrane potential
EBSS: Earle’s balanced salt solution
EndoG: endonuclease G
GSH: glutathione
GST: glutathione transferase
HPRT: hypoxanthine guanine phosphoribosyl transferase
IFN: interferon
IL: interleukin
iNOS: inducible nitric oxide synthase
LPO: lipid peroxidation
LPS: lipopolysaccharide
MAPK: mitogen-activated protein kinase
mTOR: mammalian target of rapamycin
NF-κB: nuclear factor kappa-light-chain-enhancer of activated B cells
NQO: 4-nitroquinoline-N-oxide
Nrf2: nuclear factor erythroid 2-related factor 2
PGE2: prostaglandin E2
PKA: protein kinase A
PTP: mitochondrial permeability transition pore
ROS: reactive oxygen species
TNF-α: tumor necrosis factor-alpha
